# Health Effects of Natural Environmental Radiation during Burning Season in Chiang Mai, Thailand

**DOI:** 10.3390/life12060853

**Published:** 2022-06-08

**Authors:** Narongchai Autsavapromporn, Chutima Krandrod, Pitchayaponne Klunklin, Rawiwan Kritsananuwat, Churdsak Jaikang, Kittikun Kittidachanan, Imjai Chitapanarux, Somchart Fugkeaw, Masahiro Hosoda, Shinji Tokonami

**Affiliations:** 1 Division of Radiation Oncology, Department of Radiology, Faculty of Medicine, Chiang Mai University, Chiang Mai 50200, Thailand; pitchayaponne.kl@cmu.ac.th (P.K.); kittikun.k@cmu.ac.th (K.K.); imjai.chitapanarux@cmu.ac.th (I.C.); 2 Institute of Radiation Emergency Medicine, Hirosaki University, Hirosaki 036-8564, Japan; kranrodc@hirosaki-u.ac.jp (C.K.); m_hosoda@hirosaki-u.ac.jp (M.H.); tokonami@hirosaki-u.ac.jp (S.T.); 3 Natural Radiation Survey and Analysis Research Unit, Department of Nuclear Engineering, Faculty of Engineering, Chulalongkorn University, Bangkok 10330, Thailand; rawiwan.kr@chula.ac.th; 4 Toxicology Section, Department of Forensic Medicine, Faculty of Medicine, Chiang Mai University, Chiang Mai 50200, Thailand; churdsak.j@cmu.ac.th; 5 School of Information, Computer, and Communication Technology, Sirindhorn International Institute of Technology, Thammasat University, Pathum Thani 12120, Thailand; somchart@siit.tu.ac.th; 6 Graduate School of Health Science, Hirosaki University, Hirosaki 036-8564, Japan

**Keywords:** lung cancer, natural environmental radiation, indoor radon, external dose, burning season

## Abstract

This paper presents the first measurement of the investigation of the health impacts of indoor radon exposure and external dose from terrestrial radiation in Chiang Mai province during the dry season burning between 2018 and 2020. Indoor radon activity concentrations were carried out using a total of 220 RADUET detectors in 45 dwellings of Chiang Mai (7 districts) during burning and non-burning seasons. Results show that indoor radon activity concentration during the burning season (63 ± 33 Bq/m^3^) was significantly higher (*p* < 0.001) compared to the non-burning season (46 ± 19 Bq/m^3^), with an average annual value of 55 ± 28 Bq/m^3^. All values of indoor radon activity concentration were greater than the national (16 Bq/m^3^) and worldwide (39 Bq/m^3^) average values. In addition, the external dose from terrestrial radiation was measured using a car-borne survey during the burning season in 2018. The average absorbed rate in the air was 66 nGy/h, which is higher than the worldwide average value of 59 nGy/h. This might be due to the high activity concentrations of ^238^U and ^323^Th in the study area. With regards to the health risk assessment, the effective dose due to indoor radon exposure, external (outdoor) effective dose, and total annual effective dose were 1.6, 0.08, and 1.68 mSv/y, respectively. The total annual effective dose is higher than the worldwide average of 1.15 mSv/y. The excess lifetime cancer risk and radon-induced lung cancer risk during the burning season were 0.67% and 28.44 per million persons per year, respectively. Our results substantiate that indoor radon and natural radioactive elements in the air during the burning season are important contributors to the development of lung cancer.

## 1. Introduction

According to International Agency for Research on Cancer (IARC), lung cancer (LC) is one of the leading causes of cancer mortality among both men and women worldwide [1,2]. In Thailand, LC is the second cause of incidence and mortality particularly in Upper Northern Thailand (UNT) [3,4]. Chiang Mai is the largest city in UNT and LC is one of the most common cancers for both genders as reported by World Health Organization (WHO) [5]. Multiple risk factors can cause LC in Chiang Mai, such as cigarette smoking, air pollution, and natural background radiation (e.g., radon and gamma) [3,4,6]. Cigarette smoking is the main cause of LC development, while radon (^222^Rn), the most stable isotope of radon element is identified as the second leading cause of LC and the major risk factor among non-smokers [7,8,9].

Radon (and its progeny) is the major contributor (more than 50%) of natural environmental radiation on the surface of the earth reported by WHO [7,9] and have been classified as a human carcinogen (group 1) that can cause LC by IARC [8]. It is a radioactive gas (half-life of 3.82 days), invisible, odorless, and colorless. It naturally occurs as a decay product of radium-226 (^226^Ra) and is ultimately a member of the uranium-238 (^238^U) series, found in the soil, rocks, groundwater, and air [7,9]. Approximately, 8–33% of all LC deaths worldwide are likely caused by indoor radon exposure [7,10,11,12]. Therefore, chronic exposure to radon and its decay products can induce DNA damage through chromosome alterations and double-strand breaks (DSBs), which subsequently increase the risk of LC [11,13]. Moreover, it indicates that radon and their decay products may exist in air pollutants including particulate matter (PM) with a diameter of less than 10 μm (PM_10_), smoke haze, and small dust particles, and all these elements together lead to LC development [14]. 

Lately, Chiang Mai has been annually facing adverse health impacts of airborne PM including LC and respiratory diseases during the dry season burning for over 20 years. This is because farmers burn biowaste materials from agricultural land and forest fires [15]. The highest levels of PM are seen between November and April every year and the peak tends to occur around the middle of March. Our previous study [16] indicates that the annual average indoor radon activity concentration (57 Bq/m^3^) in Chiang Mai is considered to be higher than the worldwide average (39 Bq/m^3^) and national average (16 Bq/m^3^) values [7,12,17]. An indoor and outdoor-radon activity concentration during the burning season (Mid-March) were 5.5 and 4-fold higher than the worldwide average, respectively. Therefore, it is important to elucidate the long-term measurements of indoor radon levels, particularly during the dry season burning. This paper provides the first attempt that investigates the indoor radon activity concentration and external dose from terrestrial radiation conducted between 2018 to 2020, particularly during the burning season in the Chiang Mai province. Additionally, we assessed the health risk for the potential impact of human health outcomes based on natural environmental radiation.

## 2. Materials and Methods

### 2.1. Study Area and Selection of Measurement Locations

**Figure 1:**
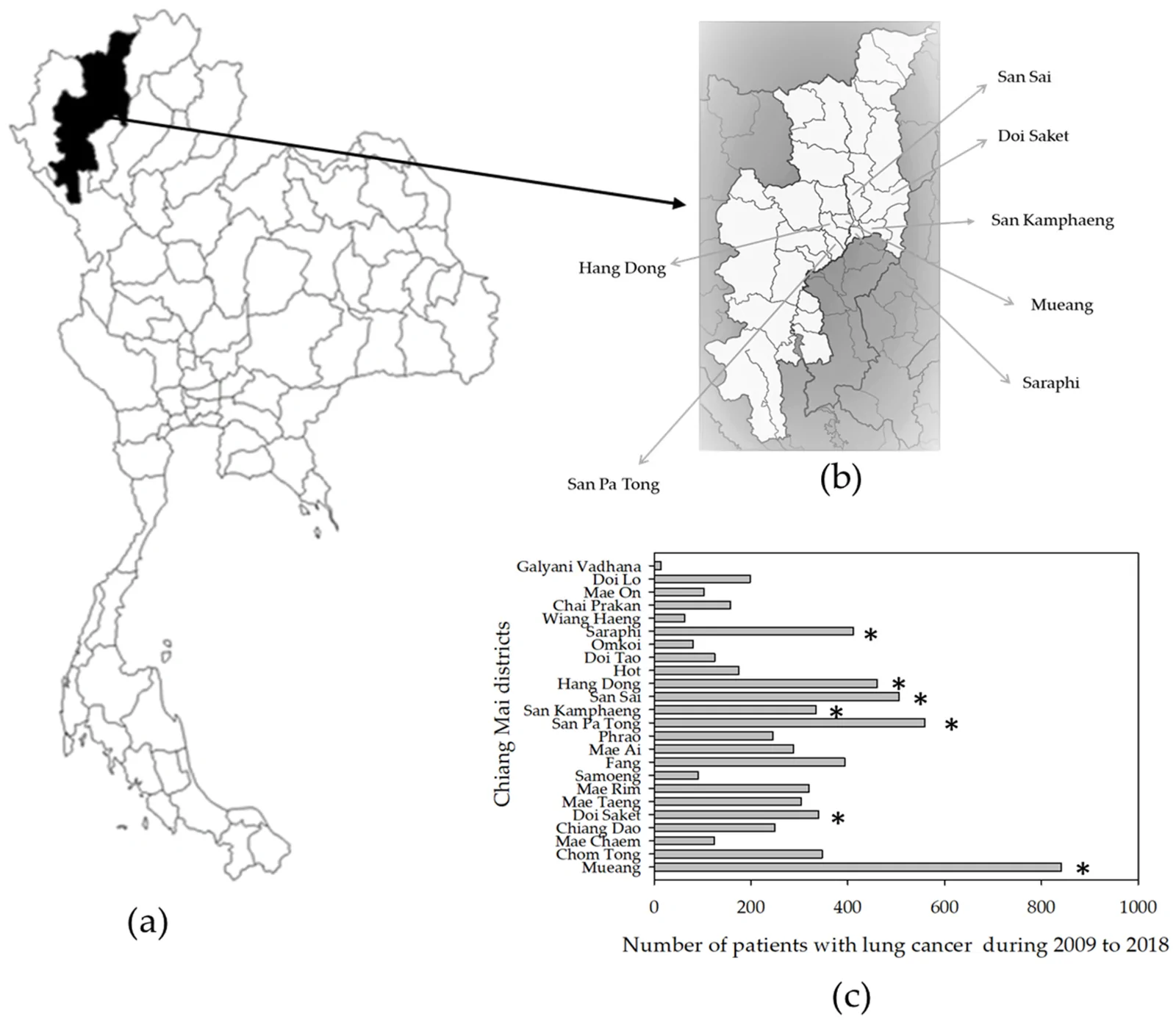
Location of measurement sites in Thailand. (**a**) Map of Chiang Mai, Thailand. (**b**) Map of Chiang Mai showing the study areas. (**c**) Number of lung cancer patients in Chiang Mai (*: study area).

Chiang Mai is the second-largest city in Thailand and the largest city in UNT. There are divided into 25 districts with a population of approximately 1.19 million residents which represents 6% of the total population in Thailand (Figure 1a). It is located on the Mae Ping River and surrounded by mountains in particular granitic rock (high background radiation area), such as Daen Lao and Thanon Thong Chai. Chiang Mai has lower humidity and a tropical climate characterized by three seasons: the winter (November–February), summer (March–May), and rainy (June–October).

This research was carried out in seven districts (high radon potential zone) located in different areas in Chiang Mai (Figure 1b). This area is affected by a high number of LC patients from 2009 to 2018 (Figure 1c). Between 2016 to 2018, indoor radon activity concentration measurements were carried out in a total of 172 randomly selected dwellings (1–5 dwellings in each subdistrict randomly depending on the district size). The districts surveyed are Mueang, Hang Dong, Saraphi, and San Pa Tong. In addition, 45 randomly selected dwellings (Mueang, Hang Dong, Saraphi, San Pa Tong, San Sai, San Kamphaeng, and Doi Saket) were selected for the study of indoor radon during the dry season burning in the period between 2018 to 2020. Most of the selected dwellings in the study area were built of cement and wood along with concrete floors.

The study encompassed fieldwork and data collection from participants by interviews (questionnaire concerning information about dwelling characteristics, family histories of LC, and lifestyle). All participants were informed of the study information about indoor radon measurements, risks, or benefits that may occur from the study. Informed consent was obtained from all participants prior to the enrollment. 

### 2.2. Radon Activity Concentration Measurement

A passive type of radon-thoron discriminative monitor (RADUET) using an α track type radon detector (CR-39) was used to measure indoor radon in the bedroom (ground floor) of selected dwellings (172 RADUET detectors) for a period of six months between 2016 and 2018 [16]. The RADUET detectors were placed away from sunlight, windows, doors, and electric devices, at a distance of 20 cm from the internal wall and a height of 100 to 200 cm from the floor as representative of human breathing inside the bedroom. At the end of the measurement, all RADUET detectors were collected, wrapped in a plastic bag, shipped, and measured at the Institute of Radiation Emergency Medicine, Hirosaki University. Briefly, CR-39 was chemically etched using a solution of 6.25 M NaOH at 90 °C for 6 h, then washed with distilled water and dried. Afterward, α particles in CR-39 were taken by digital camera and counted with an automatic reading system to evaluate the indoor radon activity concentration. 

The radon activity concentration (C) is calculated using Equation (1):

(1) $$C=\frac{\rho }{\mathrm{kt}}$$

where ρ is α particles track density corrected by background track density(track/cm^2^), k is the conversion factor from α particles track density to indoor radon activity concentration [(tracks/cm^2^/h)/(Bq/m^3^)], t is exposure time (h). It should be noted that the contribution of thoron and its progeny in this study is relatively small compared to radon and should be negligible (data not shown) [16].

In order to study the effects of biomass burning on indoor radon levels (total of 220 RADUET detectors), the experiments were performed in two sets of 45 random dwellings for periods of 12 months (replaced with a new RADUET detector every 2–3 months to cover the burning season (November–April) and non-burning season (May–October) in Chiang Mai); the first period in 2018–2019 (20 dwellings) and the second period in 2019–2020 (25 dwellings).

### 2.3. Car-Borne Survey

A car-borne survey technique is an effective method to evaluate the external radiation dose from terrestrial gamma radiation [uranium (^238^U) series, thorium (^232^Th) series, and potassium (^40^K)] in the Saraphi district which is subdivided into twelve subdistricts for a short period during the burning season from March 16–17 and 19 in 2018 using a 3-in × 3-in NaI(Tl) scintillation spectrometer (EMF-211, EMF Japan Co., Osaka, Japan) [18]. We selected the Saraphi district as one of the target areas because it is well documented that this area has a higher number of LC patients and is one of the most polluted areas in Chiang Mai [3]. The detector was installed inside a car at 1 m from the ground level and gamma radiation counting was carried out every 30 s in a moving car along the survey route with a global positioning system (the latitude and longitude for each measurement point). During the survey (a total of 821 measurement points), the car was moving at a speed of around 30–40 km/h depending on the road conditions, and the shielding factor of the car body was calculated at 18 measurement points by measurements outside and inside of the car. The methodology of the car-borne survey, the calculation of dose rates in air, and the activity concentration of natural radionuclides (^40^K, ^238^U, and ^232^Th) to absorbed dose rate in the air was followed as previously described by Hosoda et al. [19,20].

### 2.4. Health Risk Assessment

#### 2.4.1. The Annual Effective Dose (H) of Inhalation Dose

H is the total exposure of indoor radon activity concentration (and its progeny) on residents in the study area (in a year), which corresponds to the average indoor radon calculated using Equation (2), based on the United Nations Scientific Committee on the Effects of Atomic Radiation (UNSCEAR) report [21]:

H (mSv/y) = C × F × O × T × D(2)

where C is an annual average indoor radon activity concentration in the dwellings (in Bq/m^3^), F is the equilibrium factor between indoor radon (and its progeny) (0.4), O is the occupancy factor for the residential population (0.8), T is an average exposure period (24 h × 365 days = 8760 h), and D is an inhalation dose conversion factor (9 × 10^−6^ mSv/h per Bq/m^3^) [16].

#### 2.4.2. The Annual Effective Dose to Lungs (H_L_)

H_L_ was calculated using Equation (3):

H_L_ (mSv/y) = H × W_R_ × W_T_ (3)

where W_T_ (the radiation-weighting factor) is 20 for α particles and W_T_ (the tissue weighting factor for lungs) is 0.12, as recommended by the Internal Commission on Radiological Protection (ICRP) [22].

#### 2.4.3. The External (Outdoor) Annual Effective Dose (H_e_)

H_e_ was estimated using Equation (4) based on the measured absorbed dose rate in air in the Saraphi district.

H_e_ (mSv/y) = D_a_ × DCF × T × O × 10^−3^ (4)

where D_a_ is an average outdoor absorbed dose rate in air (nGy/h), DCF is dose conversion factor received by an adult (0.7 Sv/Gy), T is 8760 h and O is the occupancy factor for the residential population (0.2) [21,23].

#### 2.4.4. Excess Lifetime Cancer Risk (ELCR)

ELCR was estimated using Equation (5):

ELCR = H × DL × RF(5)

where DL is the mean of life estimated to 77 years in Thailand and RF is the risk of fatal cancer per Sievert (0.055 Sv^−1^), as reported by ICRP [24,25].

#### 2.4.5. The Number of LC Cases per Year per Million (LCC)

LCC was given according to Equation (6):

LCC= H × RFLC(6)

where RFLC is the risk factor for LC induction per million per person of 18 × 10^−6^ mSv^−1^ y as recommended by ICRP [26,27].

### 2.5. Statistical Analysis

The software Sigma Plot10 (Sigma, St. Louis, MO, USA) and Microsoft Excel were used to conduct all statistical analyses in this study. All data presented were determined based on the mean ± standard deviation (SD), median, and geometric. The Wilcoxon signed rake test was performed to test for the mean difference between two groups of data and a *p*-value of 0.05 between groups was considered to be significant.

## 3. Results and Discussion

### 3.1. Indoor Radon Activity Concentration and Health Risk Assessment Due to Indoor Radon Exposure 

The indoor radon activity concentration and health risk assessment (H,H_L_, ELCR, and LCC) in a total of 172 dwellings in four districts of Chiang Mai province (Mueang; Hang Dong; Saraphi, and San Pa Tong) were measured between 2016 and 2018 as shown in Table 1. The indoor radon activity concentration is found to be varied from 23 (Saraphi) to 209 (San Pa Tong) Bq/m^3^ (an average value of 48 ± 20 Bq/m^3^) with a geometric mean of 45 Bq/m^3^. The highest maximum value of indoor radon in the San Pa Tong district was 209 Bq/m^3^ with approximately two times higher than the reference level (100 Bq/m^3^) imposed by WHO but this value is below the ICRP reference level of 300 Bq/m^3^ [7,26]. This might be due to the difference in radioactive elements in the soil (geological condition), dwelling characteristics, and ventilation condition. The average value of indoor radon in Chiang Mai is higher compared to the worldwide average of 39 Bq/m^3^ and national average values of 16 Bq/m^3^ [7,12,17]. About 64% of dwellings in the study area exceeded the worldwide average value of indoor radon. Our finding is in agreement with the previous study (Table 2) reported that Chiang Mai has a higher radon activity concentration than in average obtained by national and worldwide.

As the risk of individual LC development increases with duration and exposure to indoor radon, it is very pivotal to estimate the effect on human health from long-term exposure to indoor radon exposure [6,11]. Table 1 shows the estimated health risk assessment of indoor radon exposure to residents in the study area. The total values of H were calculated between 0.6 to 5.3 mSv/y (data not shown) with an average of 1.2 mSv/y. The average H is less than the action level limit of 3–10 mSv/y, as recommended by ICRP [30]. However, the average H is found to be higher than the worldwide average of 1.15 mSv/y (inhalation dose), while the average H_L_ is 2.9 mSv/y [21]. This value is higher than the worldwide average due to the stressful effects of the α particle on the lungs [6]. To consider the risk of LC due to indoor radon exposure, ELCR is used to predict the probability of cancer development by residential radon over a lifetime. The average ELCR for indoor radon exposure in the study area was 0.51%, which is lower than the action level of 1.3%, and due to indoor radon levels of 148 Bq/m^3^ as recommended by the United States Environmental Protection Agency (USEPA) [31]. However, this value is higher than the worldwide average of 0.145%, which may be related to the high radiation area in Chiang Mai [10,32]. Therefore, LCC average value in Chiang Mai caused by radon exposure was estimated to be 21.6 per million people per year. This value is lower than the limit range between 170 to 230 per million persons per year as reported by ICRP [30]. Based on the estimated values, our data show that the impact of health risk for LC development received by residents in the study area is related to chronic exposure to indoor radon. To this end, our future work will focus on the investigation of long-term indoor radon measurements with a larger sample size. 

### 3.2. Indoor Radon Activity Concentration and Health Risk Assessment during Burning- and Non-Burning Seasons

Long-term exposure to natural environmental radiation and outdoor air pollution may be associated with an increased risk of LC development [33]. Lately, Chiang Mai is facing the highest air pollution in the world, caused by the open burning of biomass during the harvest season. Despite biomass burning being important; this condition causes Chiang Mai to have a high level of radon in UNT and Thailand [10]. To our knowledge, there is little understanding of the relationship between indoor radon exposure and air pollution during the burning season in Chiang Mai, which can affect LC development and other diseases [7]. In this study, indoor activity concentration and health risk assessment were recorded in 45 dwellings in seven districts in the Chiang Mai province (Mueang (n = 6), Hang Dong (n = 7), Saraphi (n = 21), San Pa Tong (n = 5), San Sai (n = 2), San Kamphaeng (n = 2) and Doi Saket (n = 2)) during burning- and non-burning seasons between May 2018 and October 2020 are reported in Table 3. 

**Figure 2:**
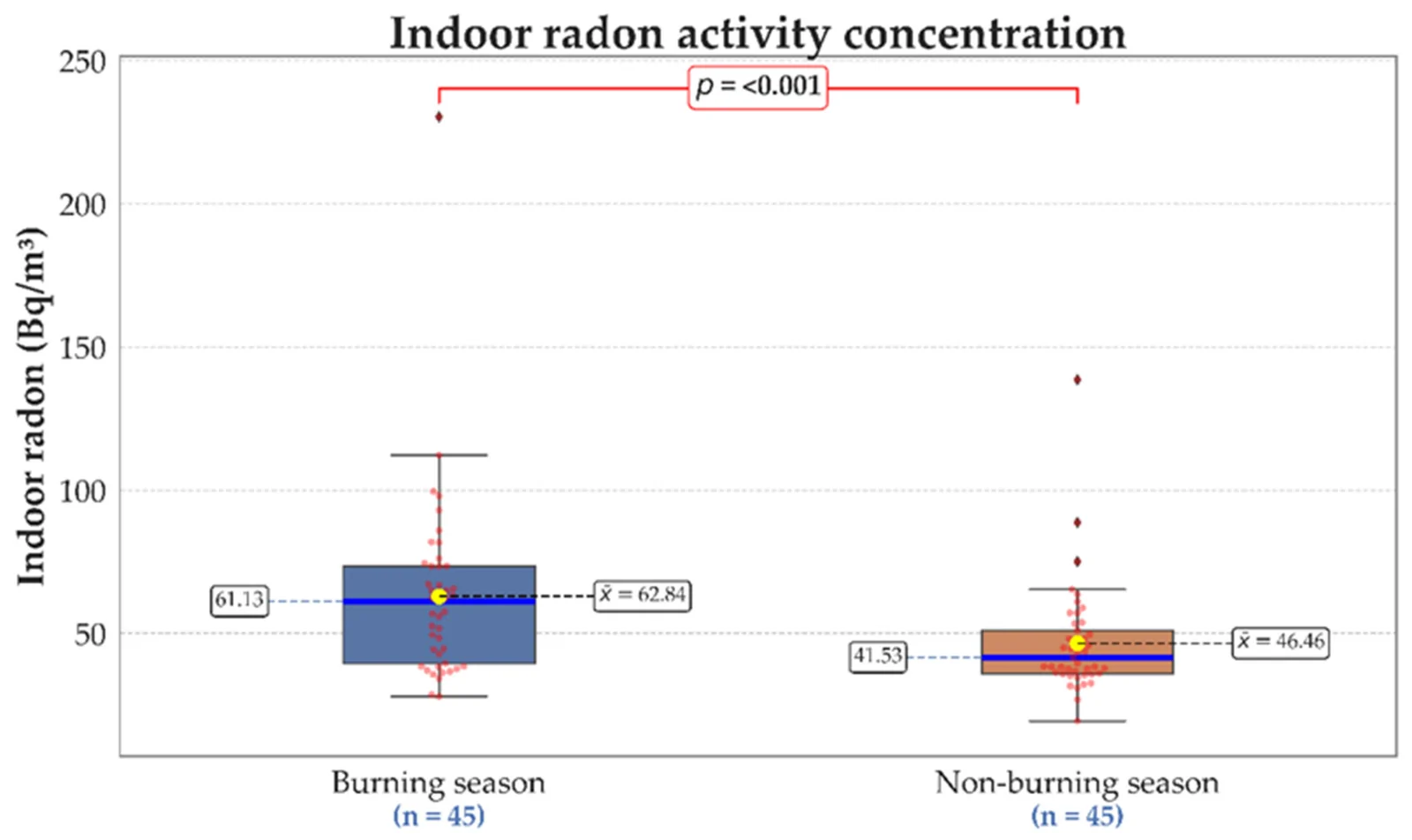
Variation of indoor radon activity concentration during burning and non-burning seasons in seven districts of Chiang Mai during 2018–2020.

As can be seen in Table 3, the annual indoor radon activity concentration measurement ranged from 19 to 230 Bq/m^3^ with an average value of 55 ± 28 Bq/m^3^ (geometric mean is found to be 50 Bq/m^3^). The highest indoor radon activity concentration was observed in the San Pa Tong district (data not shown). Overall, 42.2% of dwellings presented indoor radon activity concentration comparatively higher than the world average value and 2.2% had a value above 100 Bq/m^3^. This clearly suggests that the annual average value of indoor radon in Chiang Mai is greater than the national and worldwide average value. Interestingly, a comparison result (Table 3 and Figure 2), shows a significant statistical difference (*p* < 0.001) between indoor radon activity concentration during burning-and non-burning seasons. The average indoor radon level during the burning season (63 ± 33 Bq/m^3^ with a geomean of 57 Bq/m^3^) was found to be higher than those measured in the non-burning season (46 ± 19 Bq/m^3^ with a geomean of 44 Bq/m^3^). The difference in the radon level during biomass burning season may be due to high levels of natural background radiation in air pollution, high levels of radioactive elements in the soil, climatic parameters (such as high concentrations of radon in the winter burning season), home ventilation and building materials [34,35,36]. 

In estimating human health risk due to indoor radon exposure (and its progeny) during burning- and non-burning seasons (Table 3), H values in the study area during burning-and non-burning seasons were found to be 1.6 and 1.2, respectively, (with varies from 0.5 to 5.8 mSv/y, data not shown), with an average of 1.4 mSv/y. The estimated average H during burning- and non-burning seasons and average annual H are higher when compared with a worldwide average value of 1.15 mSv/y [21]. The calculated H_L_ due to indoor radon exposure during burning-and non-burning seasons were 3.8 and 2.8, respectively, with an average of 3.3 mSv/y. These results are higher than the action level by ICRP [30]. The ECLR attributable to residential radon during burning-and non-burning seasons were 0.67 and 0.5%, respectively, with an average value of 0.58%. All estimated values in the present data were lower than the action level reported by USEPA [31]. However, these values are higher than average worldwide [32]. The radon-induced LC during burning-and non-burning seasons were 28.44 and 21 per million people per year, respectively. While the LCC average of 24.84 per million people per year is lower than the range recommended by ICRP [30]. All together, these findings also show a significant difference (*p* < 0.05, data not shown) in all human health risk assessments on residential radon exposure (H, H_L_, ECLR, and LCC) between burning-and non-burning seasons. Therefore, this comparison indicates the potential risk of natural background radiation during the burning season to human health; hence it can be a major public health problem for the UNT of Thailand due to chronic exposures to radon (and its progeny) along their lifetime. 

To the best of our knowledge, our study is the first attempt in dealing with long-term indoor radon measurements within a human health risk assessment in the Chiang Mai province during the burning season. However, the lack of measurement of natural radioactivity concentrations such as ^40^K, ^232^Th, and ^238^U present in dust particles in the air, and outdoor radon activity concentration in the ambient air during burning- and non-burning seasons is the limitation in this study. Additional research is needed to obtain more detailed results.

### 3.3. External Radiation Dose and External Annual Effective Dose Estimation from Natural Environmental Radiation during burning Season

**Figure 3:**
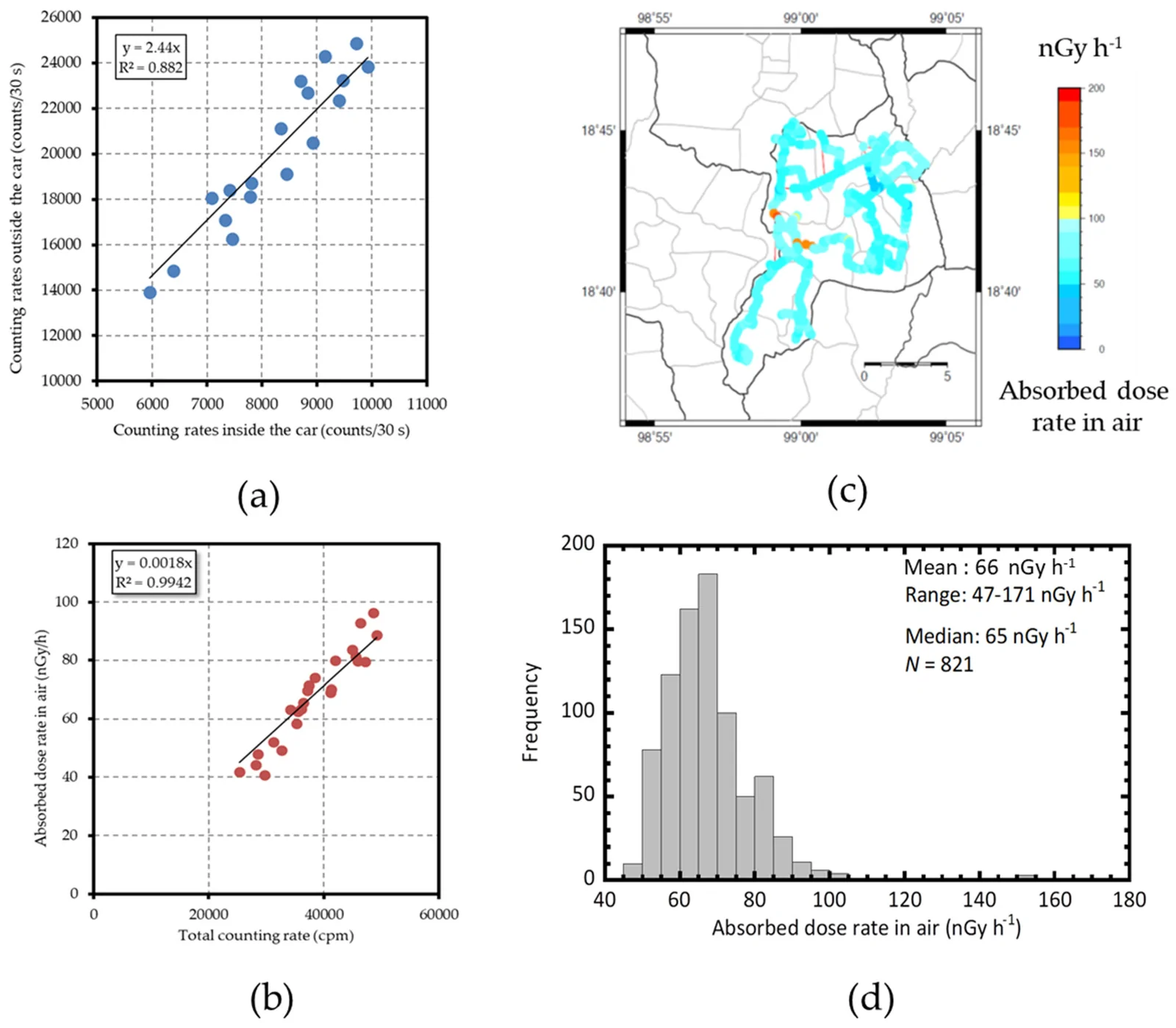
Map of outdoor gamma dose rates in the air in Saraphi district during the dry season burning measured in 2018. (**a**) Correlation between count rates outside and inside the car. (**b**) Correlation between air karma and total count rate outside the car. (**c**) Map showing the survey route and distribution of air kerma rate. (**d**) Absorbed dose rate in air in Saraphi district.

For a further understanding of the health effects of natural environmental radiation during the burning season in Chiang Mai, an external dose of terrestrial radiation for residents living in the Saraphi area was obtained by car-borne measurement during the peak of the dry season burning in 2018 (16, 17 and 19 March). The survey route consists of twelve subdistricts of Saraphi and variations of external radiation dose in the air (outdoor absorbed gamma dose rates) are shown in Figure 3c. The shielding factor (Figure 3a) and dose conversion factor (Figure 3b) were determined as 2.44 and 0.0018 nGy/h, respectively. 

A total of 821 measurement points were collected in the study areas. Figure 3c shows the outdoor gamma dose rates range from 47 to 171 nGy/h with an average value of 66 nGy/h (median value of 65 nGy/h). This average value was found to be higher than the world average value of 59 nGy/h as reported by UNSCEAR [21]. This study shows that a high outdoor gamma dose rate in air is an important contribution to external radiation dose in Chiang Mai during the dry season burning. In addition, the average gamma dose rate in air in Chiang Mai, UNT was higher than in other parts of Thailand Western (44 nGy/h), Eastern (35 nGy/h), and Southern (42 nGy/h), which may be related to high activity radioactivity area on the UNT of Thailand [37].

Furthermore, airborne gamma-ray spectrometry measurement was carried out to determine the radionuclides activity concentration contributing to natural environmental radiation during the burning season. Table 4 represents the activity concentration of ^40^K, ^238^U, and ^232^Th in soil, and the absorbed dose rate in the air 1 m above the ground was measured at 24 points in the Saraphi district. The contribution to absorbed dose rate in the air of ^40^K, ^238^U, and ^232^Th ranged from 13% to 27%, 30% to 41%, and 34% to 48% with an average value of 22%, 36%, and 42%, respectively (data not shown). As displayed in Table 4, the average activity concentration of ^40^K, ^238^U, and ^232^Th were 346 ± 90, 56 ± 16 and 43 ± 14 Bq/kg, respectively. Based on this result, the activity concentration of ^238^U and ^232^Th were higher than the worldwide average values of 35 and 30 Bq/kg, respectively [21].

These findings suggest that ^238^U and ^232^ Th-series elements are the main sources of external natural radiation exposure during the burning season in the study area. High activity concentrations of ^238^ U and ^232^ Th can be explained by the geometrical environment (such as granites) and the mechanisms of soil information [19,20,38,39]. However, it should be noted that 38% of measurement points (n = 9) have an activity concentration of ^40^K above the worldwide average value of 400 Bq/kg and were found to be a minor contribution to the total absorbed dose rate in the air [21]. Further investigation of radionuclides activity concentration in this study area is needed to confirm these results.

With regards to the health risk assessment, the mean, minimum and maximum values of external (outdoor) annual effective dose (H_e_) were estimated to be 0.08, 0.06, and 0.21 mSv/y, respectively (data not shown). The average H_e_ value of this study area was higher than the worldwide average value of 0.07 mSv/y, as reported by UNSCEAR [21]. Therefore, the outcome of radiation dose assessment indicates the relevant effects of natural environmental radiation during the dry burning season on human health.

## 4. Conclusions

We have presented our first study that provides an understanding of the impacts of biomass burning on natural environmental radiation in the Chiang Mai province, particularly indoor radon exposure and external dose from terrestrial radiation. The findings show indoor radon activity concentration (63 ± 33 Bq/m^3^) and external dose from terrestrial radiation (66 nGy/h) during the burning season was higher than the national and worldwide average value. The activity concentration of ^238^U and ^232^Th found in the soil of area studies during the burning season was higher than the worldwide average value. The estimated value of effective dose due to exposure to indoor radon (and its progeny), external (outdoor) effective dose, and the total annual effective dose received by Chiang Mai residents were 1.6, 0.08, and 1.68 mSv/y, respectively. The total annual effective dose is higher than the worldwide representative value of 1.15 mSv/y. The excess lifetime cancer risk was found to be 0.67%, which is higher than the worldwide average. The radon-induced LC risk during the burning season presents a value of 28.44 per million persons per year. With all results obtained from the fieldwork, indoor radon (and its progeny) and terrestrial radiation represent the major contributions of human exposure to natural radiation during the dry season burning and may increase the possibility of LC developing in their lifetime. Future research related to natural environmental radiation and air pollution during the burning season is required to confirm these findings.

## Figures and Tables

**Table 1:** Indoor radon activity concentration and the potential risk of lung cancer to residents of Chiang Mai, Thailand.

	Mueang	Hang Dong	Saraphi	San Pa Tong	Total
No. of dwellings	48	25	83	16	172
Max (Bq/m^3^)	99	65	100	209	209
Min (Bq/m^3^)	24	33	23	31	23
Mean ± SD (Bq/m^3^)	47 ± 17	43 ± 10	49 ± 16	50 ± 43	48 ± 20
Geometric mean (Bq/m^3^)	45	42	46	43	45
No. of Dwellings >39 Bq/m^3^	28	15	60	7	110 (64%)
No. of Dwellings >100 Bq/m^3^	0	0	0	1	1 (0.6%)
H (mSv/y)	1.2	1.1	1.2	1.3	1.2
H_L_ (mSv/y)	2.9	2.6	3.0	3.0	2.9
ELCR (%)	0.51	0.47	0.52	0.53	0.51
LCC (×10^−6^)	21.6	19.8	22.1	22.7	21.6

Abbreviations: SD, standard deviation; H, annual effective dose; H_L_, annual effective dose to lungs; ELCR, excess lifetime cancer risk; LCC, the number of LC cases per year per million.

**Table 2:** Comparison of the average indoor radon activity concentration in Chiang Mai.

Study Areas (Districts)	No. of Detectors/Period	Indoor Radon (Bq/m^3^)	Ref.
Saraphi	50/99 days	21	[28]
Mueang, Hang Dong, Saraphi and San Pa Tong	110/1 year	57	[16]
Doi Saket	30/4 months	53	[29]
Not available	46/3 months	110	[10]
Mueang, Hang Dong, Saraphi and San Pa Tong	172/6 months	48	This study

**Table 3:** Indoor radon activity concentration and potential risk of lung cancer during burning and non-burning seasons to Chiang Mai residents during 2018–2020.

	Burning Season	Non-Burning Season	Total
No. of dwellings	45	45	45
Period (months)	6	6	12
Max (Bq/m^3^)	230	139	230
Min (Bq/m^3^)	28	19	19
Mean ± SD (Bq/m^3^)	63 ± 33	46 ± 19	55 ± 28
Median (IQR) (Bq/m^3^)	61 (35)	42 (16)	48 (27)
Geomean (Bq/m^3^)	57	44	50
No. of Dwellings >39 Bq/m^3^	34	24	19 (42.2%)
No. of Dwellings >100 Bq/m^3^	2	1	1 (2.2%)
H (mSv/y)	1.6	1.2	1.4 ± 0.3
H_L_ (mSv/y)	3.8	2.8	3.3 ± 0.7
ELCR (%)	0.67	0.5	0.58 ± 0.12
LCC (×10^−6^)	28.44	21	24.72 ± 5.26

Abbreviations: SD, standard deviation; IQR, interquartile range; H, annual effective dose; H_L_, annual effective dose to lungs; ELCR, excess lifetime cancer risk; LCC, the number of LC cases per year per million.

**Table 4:** The measured activity concentration of ^40^K, ^238^U, and ^232^Th and absorbed dose rate in air in Saraphi district during the dry season burning.

Point No.	Location		Activity Concentration (Bq/kg)			Absorbed Dose Rate in Air (nGy/h)
	Latitude (°)	Longitude (°)	^40^K	^238^U	^232^Th	
1	18.6659	98.9742	234	30	21	42
2	18.633	98.9637	306	43	30	52
3	18.6514	98.9698	372	42	37	59
4	18.686	98.9954	404	44	40	63
5	18.6521	99.0001	298	35	28	48
6	18.6754	99.0043	398	46	46	69
7	18.6969	98.9956	437	69	38	70
8	18.7048	98.9969	416	70	51	80
9	18.6894	99.0134	439	65	51	80
10	18.6932	99.0274	451	76	62	89
11	18.6862	99.0573	330	46	36	63
12	18.6821	99.0387	147	30	26	41
13	18.7054	99.0384	413	77	68	93
14	18.7169	99.0263	173	34	30	44
15	18.72	99.0633	297	88	72	96
16	18.6986	99.0591	294	69	61	81
17	18.745	99.0405	338	59	37	66
18	18.7416	99.0568	396	65	38	74
19	18.7367	99.0661	335	72	54	84
20	18.7203	99.0136	173	49	29	49
21	18.739	99.0214	409	59	44	71
22	18.7288	98.9943	438	66	55	80
23	18.7499	98.9997	427	54	41	70
24	18.7158	99.004	376	47	37	63
Average			346 ± 90	56 ± 16	43 ± 14	68 ± 16

## Data Availability

Not applicable.
